# Environmental and Occupational Exposures in Immigrant Health

**DOI:** 10.4137/EHI.S847

**Published:** 2008-09-23

**Authors:** Pracha P. Eamranond, Howard Hu

**Affiliations:** 1 Harvard School of Public Health, Boston, MA; 2 Division of General Medicine and Primary Care, Beth Israel Deaconess Medical Center, Harvard Medical School, Boston, MA; 3 Department of Medicine, Brigham and Women’s Hospital, Harvard Medical School, Boston, MA; 4 Department of Environmental Health Sciences and Department of Internal Medicine, University of Michigan Schools of Public Health and Medicine, Ann Arbor, MI

**Keywords:** immigrant, occupational, environmental, exposure, hazard

## Abstract

Immigrants comprise vulnerable populations that are frequently exposed to a multitude of environmental and occupational hazards. The historical context behind state and federal legislation has helped to foster an environment that is particularly hostile toward caring for immigrant health. Current hazards include toxic exposures, air and noise pollution, motor vehicle accidents, crowded living and work environments with inadequate ventilation, poor sanitation, mechanical injury, among many others. Immigrants lack the appropriate training, materials, health care access, and other resources to reduce their exposure to preventable environmental and occupational health risks. This dilemma is exacerbated by current anti-immigrant sentiments, miscommunication between native and immigrant populations, and legislation denying immigrants access to publicly funded medical care. Given that current health policy has failed to address immigrant health appropriately and political impetus is lacking, efforts should also focus on alternative solutions, including organized labor. Labor unions that serve to educate workers, survey work environments, and defend worker rights will greatly alleviate and prevent the burden of disease incurred by immigrants. The nation’s health will benefit from improved regulation of living and workplace environments to improve the health of immigrants, regardless of legal status.

## Introduction

We have been a country of immigrants since the beginning of U.S. history. The immigrant population within the U.S. continues to rise beyond previous records, reaching an estimated 34.2 million (12%) in the 2004, 62% of whom are not citizens.^1^ Immigrants have a higher rate of poverty (16.8%) compared to the U.S. native population (11.8%) and comprise 22% of the nation’s uninsured population.^2^ Immigrants comprise an underprivileged population that continues to be neglected from various standpoints, including environmental and occupational health exposures. Disparities in immigrant health are exacerbated by lack of adequate health care access and culturally-inappropriate health care. Observers in some states have asserted that immigrants place an extra burden on health care systems, which may explain the low priority given to immigrant health care.^3^ However, immigrants contribute as much as $10 billion/year to the U.S. economy and pay taxes in excess of $80,000 per capita more than the value of government services received over their lifetimes.^4^ Overestimates of immigrant utilization of resources and underestimates of immigrant contribution to the U.S. economy may give rise to a general reluctance to provide health services for immigrant populations.

Prior to discussing environmental and occupational health, let us look at the past and present context of health care for immigrants in the U.S. The Personal Responsibility and Work Opportunity and Reconciliation Act (PRWORA) of 1996 deemed all legal immigrants ineligible for non-emergency Medicaid services. Although this may have led to modest savings in federal welfare expenditures, an unintended result has been a substantial surge of uncompensated costs for acute-care facilities from both documented and undocumented immigrants. In part, this is due to uninsured immigrants who cannot access primary care follow-up, obtain necessary medicines or equipment, or cannot be transferred to long-term care facilities.^5^ It is difficult to compare current uncompensated hospital care for immigrants with those medical expenditures prior to PRWORA. One study of 300 hospitals demonstrated that the median uncompensated cost per hospital after PRWORA for immigrants was 2.6 million dollars annually.^6^ Other studies have shown that channeling immigrants into acute-care facilities to receive medical care is likely much less cost-effective than providing preventive services to all immigrants.^7^,^8^

The 1996 Illegal Immigration Reform and Immigrant Responsibility Act (IIRAIRA) included some of the most drastic measures ever taken against illegal immigration. The legislation made illegal immigrants ineligible for federal, state, and local public benefits.^9^ In conjunction with PRWORA, this legislation bars most future immigrants from applying for federal public benefits for the first five years in the U.S. Even after the initial five-year period, access to publicly funded medical care for qualified aliens would be severely restricted. IIRAIRA and PRWORA have had a ripple effect on children of immigrants as well. Although many children of immigrant parents are citizens, these children are more likely to be uninsured compared to children of native-born parents.^10^

Support for Proposition 187, a statewide immigration reform initiative in California, heralded some of strongest and most persuasive arguments against using public funds for immigrant health care. Advocates argued that ‘an invasion of illegal aliens’ was bankrupting California and declared, ‘While our own citizens and legal residents go wanting, those who choose to enter our country illegally get royal treatment at the expense of the California taxpayer.’^11^ In 1994, California approved Proposition 187, which proposed to deny publicly funded health care, social services, and education to illegal immigrants. Proposition 187 further required that if a facility ‘determines or reasonably suspects’ that a patient is an illegal immigrant, it must report the patient to the Immigration and Naturalization Service, the state attorney general, and the state director of health services.^12^ Although the major provisions of the proposition have not been implemented due to a U.S. district court injunction, deaths of illegal immigrants have been attributed to delays in seeking medical care because of fear of deportation.^13^ Due to the paucity of information available on health outcomes of immigrants who do not seek care, it is difficult to gauge the impact of anti-immigrant legislation on immigrant health. Currently, investigating health needs and providing health care for immigrants is taking a back seat to methods determining legal status of immigrants as well as keeping illegal immigrants out of the U.S.

For example, states have been struggling to comply with the Centers for Medicare and Medicaid Services (CMS) attempts to limit illegal immigrant Medicaid coverage. Stricter requirements for proving citizenship are ironically hindering the most vulnerable citizens from obtaining coverage.^14^ Foster children, the homeless, victims of natural disaster, and others who do not have access to a birth certificate are among those legal citizens that cannot come up with the required documentation. Furthermore, recent immigrants are more likely to have documentation to prove their legal status than some U.S.-born citizens.

Xenophobic attitudes are easily seen outside of the U.S. as well, particularly in developed countries. For example, foreigners and refugees in Germany are ‘constantly reminded in everyday life that they are not Germans and that they do not belong.’^15^ The language applied to immigrants through mass media outlets likely plays an important role in the fear generated in both the immigrant and native-born populations. This rhetoric includes wording such as immigrants *pouring* across borders, arriving in *swarms*, and *overrunning* communities.^16^ In an effort to deter illegal immigration, the Australian government declared: “We will choose who comes to these shores and the circumstances under which they come.”^17^ The tabloid press in the U.K. provides a stark example of fear-instilling mass media: “Illegal immigration. Radical Islam. Terrorism. Crime. Disease. An overstretched health service. And to make it all worse: falling property prices.”^18^ Even though these and other wealthy nations are actively recruiting skilled immigrants such as nurses, doctors, engineers and other scientists to offset their own labor shortages,^19^ these immigrants are victims of ‘othering’ and may remain outsiders within the communities they live.^20^

Although other developed countries have certainly had problems with immigrant issues, including health care,^21^–^24^ most national healthcare programs have served to buffer the problems raised by immigrants who arrive without health care coverage. For example, contrary to U.S. hospitals suffering the brunt of uncompensated immigrant health utilization, hospitals in Spain report lower resource use per hospitalization among immigrants compared to Spanish natives.^25^ Despite U.S. health expenditures exceeding more than one trillion dollars per year (more than any other country), the U.S. remains the only economically developed country that lacks a national healthcare program ensuring universal access to care.^26^ Denmark, for example, has prioritized preventive measures and public health initiatives concerning ethnic minorities. Due to lack of outcome data, there is debate whether national health coverage in the U.S. would lead to comparable quality of care. However, there is some evidence in Denmark showing that health care utilization, particularly with hospital stay and inpatient care, are equivalent among immigrants versus Danish-born patients.^27^ In a setting where health indicators are excellent,^28^ the Danish are focused on identifying why utilization patterns differ between immigrants groups while still ensuring equity in access to healthcare services for immigrants.^29^

**Figure 1.**
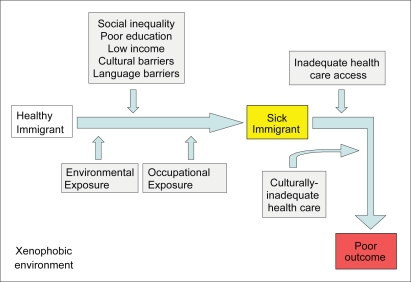
Immigrant health disparities.

Although some barriers to immigrant health are seemingly difficult to address from a health policy perspective, many environmental and occupational exposures are easily identified and can be readily corrected. There are inherent differences between immigrants and native populations that may appear daunting from an epidemiological and clinical standpoint. These include nutrition, health beliefs, religion, language, and other distinct cultural differences. Understanding these differences will ultimately improve health care for all populations, including immigrants. Figure 1 shows a brief schematic of how various forces can lead to poor health outcomes, particularly for immigrants. The following discussion of environmental and occupational risks for immigrants focuses on obvious disparities that do not require a profound understanding of cultural issues and can be addressed from a policy perspective, if our public will demands it.

## Environmental Exposures

In terms of environmental exposures, relatively little is known about risks incurred by immigrant populations. In Massachusetts, the prevalence of elevated blood lead levels among immigrant children was greater than twice as high as in U.S.-born children.^30^ A large percentage of these refugees acquired elevated levels after arrival to the U.S. More prevalent environmental exposures, such as exhaust and noise from motor vehicle traffic, dust from construction projects, motor vehicle accidents, and lack of open/green space, are among the environmental hazards that plague immigrants in Boston Chinatown.^31^ Since housing conditions and environmental effects of traffic directly influence health, the Chinatown population (composed mostly of first-generation immigrants) is at particular risk to environmental factors. Chinatown is the only neighborhood in Boston that is located at the juncture of two major highways that account for one quarter of a million vehicle trips daily. Among the environmental exposures listed above, noise and air pollution are associated with elevated blood pressure, cholesterol levels, and impairment of reading and language skills in children.^32^ Components of vehicle exhaust and particulate matter have been associated with a variety of acute and chronic conditions, including headaches, eye conditions, and asthma.^33^ A study in Boston Chinatown has suggested that carbon monoxide levels could exceed federal limits.^34^ Residents in Chinatown are largely unaware that the environmental exposures above, including lead, are hazardous to their health.^8^

A recurring theme in immigrant discrimination is the high risk attributed to immigrants based solely on infectious disease epidemiology from their country of birth rather than high-risk environments here in the U.S. For example, the medical community has long attributed tuberculosis outbreaks in large part to socioeconomic conditions such as poor nutrition, overcrowding and inadequate housing for non-immigrant populations. However socioeconomic factors are not applied to immigrants in the same way. In fact, some public health workers in New York City believe that foreign culture of immigrants prevents effective tuberculosis treatment.^35^ While the number of U.S.-born cases of tuberculosis has declined since 1992, immigrant cases of tuberculosis have increased. China has been the largest contributor of foreign-born tuberculosis, and Chinese laborers have been noted to be disproportionately affected among the general Chinese population. This ultimately suggests one link between environmental and occupational exposures that lead to disease transmission.

## Occupational Exposures

With regard to occupational exposures, immigrants participate in high-risk occupations including agriculture, sweatshops, industry, and construction. Immigrants working as seasonal farmworkers are likely to suffer from occupational exposures including pesticides, sun, poor field sanitation, and mechanical injuries. Part of this problem may be due to unsafe practices learned abroad that go uncorrected here in this country. Although a large proportion of U.S. agricultural production is reliant on the immigrant labor force, little is done to educate these workers to reduce risk of occupational harm. In a study of adolescent Latino farmworkers, most of who were from Mexico, 21.6% reported working with agricultural chemicals (mixing/applying).^36^ However, few ever reported receiving pesticide training. Of note, an estimated 7% of the Latino agricultural workforce is composed of adolescents. Chronic organophosphate pesticide exposure among immigrant Hispanic farmworkers negatively impacted neurobehavioral performance, even at low levels of exposure.^37^ Little is known about migrant populations such as these due to a variety of factors including concerns of methodological difficulties in epidemiological studies, mobile nature of immigrant workers, and communication barriers.

Another recent and stunning example of the treatment of immigrant workers occurred in the aftermath of September 11th. In a study^38^ evaluating day laborers working in cleaning operations around Ground Zero, the vast majority was Hispanic, mostly from Colombia and Ecuador. Most did not speak English, did not have health insurance, and did not receive training in working with hazardous materials. Many participants were hired off the streets and were generally not provided with respirators or any personal protective equipment. Nearly all of the examined workers reported irritation of the airways (cough, sore throat, chest tightness) and/or systemic symptoms (headaches dizziness, sleep disturbances). Most participants with 4–8 weeks of dust exposure reported no or little improvement after cessation of work.

In a cross-sectional study of Asian immigrant garment workers, 16% had nerve entrapment syndromes and 99% had a diagnosed strain or sprain of the spine or upper extremities.^39^ This population did not file workers’ compensation claims for fear of reprisal or lack of knowledge. Among a survey of Cambodian and Laotian immigrants, 40% reported working in electronics and computer assembly, being exposed to soldering fumes, inadequate ventilation, prolonged sitting or standing, awkward postures, long hours, and pressure to produce quickly.^40^ A quarter of those employed held temporary jobs. Less than a third of respondents knew about workers’ compensation.

Among non-agricultural immigrant Latino workers, the average occupational injury rate was 12.2 per 100 full-time workers, compared to an expected 7.1 injuries per 100 full-time workers in the U.S. population.^41^ In this population, safety training was provided to a minority or workers and was not delivered in Spanish or indigenous languages, despite most respondents reporting limited or no proficiency in English. Only 20% of participants had employment-related health insurance (compared to 28% of U.S. workers of similar low-wage jobs), almost 60% did not have workers’ compensation (compared to almost 75% of all U.S. working adults).

Construction workers in particular suffer the largest number of fatal occupational injuries of any industry sector. With the dramatic increase in the Hispanic population due to immigration from 1990 to 2000 (5 to 12%), there has also been a concomitant increase in occupational fatalities of Hispanic construction workers, nearly twice as likely to be killed compared to their non-Hispanic counterparts.^42^ Latino construction workers are at particular risk given the hazardous tasks that they perform, many have a low level of English, and have little training in their native language. Many young Latino workers have low levels of English. The median training time was only one hour and only 24% reported receiving written training material.^43^

## Translating Research into Regulation

Although many of the disparities described in this review can be partially addressed through broader national healthcare coverage, there are many other feasible solutions that would prevent poor outcomes from environmental and occupational exposures. For example, a national environmental and occupational health surveillance system would identify health disparities and bring them to the forefront of health policy agendas. As the current administration pushes for legislation to create a guest worker program, those programs should include some increased federal oversight of living and working conditions. A federal oversight committee could implement measures such as the proper living conditions to prevent overcrowding or adequate safety training and distribution of safety equipment offered to all employees in their respective languages. Much has yet to be done to protect environmental and occupational rights of immigrant populations.

As there is little current political impetus to enact legislation to protect immigrants from environmental and occupational hazards, the burden may be best addressed through local entities such as labor representation. Although membership has declined for both native and foreign-born workers between 1996 and 2003, foreign-born workers are still underrepresented (10%) compared to native workers (13%).^44^ Unions have the potential to offer a multitude of opportunities, from education to support for political action. Labor unions remain one of the few organizations to join workers to monitor workplace conditions, defend occupational rights, and organize to create new regulations for future prevention. Worker centers are particularly vital to improving support for low-wage workers, particularly immigrants. As of May 2005, there were 137 worker centers, 122 of them identified as immigrant worker centers.^45^ Key services provided by worker centers include worker rights education, English classes, and legal representation. Worker centers also play a key role in advocacy for improvement of harmful work conditions through research and lobbying for new legislation.

The purpose of this commentary is not to give a complete list of solutions, but to raise awareness and advocacy given immigrants comprise vulnerable populations and are potentially less able to demand proper protections from environmental and occupational hazards. Public health research is needed to further elucidate disparities and implement interventions to improve the health of immigrants. Perhaps one solution should be less effective, but less expensive, interventions such as those proposed by the Kennedy Krieger lead paint abatement study.^46^ Critical, dogmatic stances that perpetuate the status quo should not prohibit research and health care from reaching immigrant populations.

## Conclusion

In this age of globalization, people are traveling all over the world to live and work. This is particularly true for the U.S. Unnecessary health disparity is created through lack of appropriate health care infrastructure for immigrant populations, a xenophobic environment that precludes communication, and lack of environmental/occupational education and protection of immigrant rights. Immigrant health disparities have been and will increasingly become an important human rights dilemma of our time. Addressing immigrant health disparities will require epidemiologic research that is specific to this group, community-based environmental education programs, organized labor movements, improved health care access, culturally competent care, and changes in health policy that are designed to address inequities in exposures to environmental and occupational hazards.
